# Perceived emotional intelligence and individual resilience in a cross-sector sample of employees

**DOI:** 10.3389/fpsyg.2026.1901514

**Published:** 2026-10-26

**Authors:** Marcos Martinez-Jurado, Maria P. Egea-Romero, Elena Saiz-Clar, Amable Cima-Muñoz, Esther Rincon

**Affiliations:** 1 Departamento de Psicología y Pedagogía, Facultad de Medicina, Universidad San Pablo CEU, CEU Universities, Alcorcón, Spain; 2 Psycho-Technology Lab, Universidad San Pablo CEU, CEU Universities, Alcorcón, Spain; 3 Departamento de Medicina y Cirugía, Universidad Cardenal Herrera-CEU, CEU Universities, Alcorcón, Spain; 4 Instituto Universitario de Estudios de las Adicciones (IEA-CEU), Universidad San Pablo CEU, CEU Universities, Alcorcón, Spain

**Keywords:** employees, perceived emotional intelligence, positive organizational psychology, resilience, workplace

## Abstract

**Introduction:**

Emotional resilience is a key psychological resource for maintaining well-being and adaptation in the workplace and is closely associated with perceived emotional intelligence. However, understanding of this relationship among working populations remains limited. The aim of this study was to examine the relationship between perceived emotional intelligence and resilience and to explore their associations with sociodemographic and occupational variables.

**Methods:**

Using a cross-sectional observational design, the study included 103 employees from 11 organizations across seven sectors in Spain. Participants completed an online questionnaire including the Spanish-adapted Wong and Law Emotional Intelligence Scale (WLEIS-S), the Resilience Scale-14 (RS-14) and sociodemographic and occupational variables.

**Results:**

A positive and statistically significant association was found between perceived emotional intelligence and resilience (*r* = 0.762, *p* < 0.001). All four dimensions of perceived emotional intelligence were also positively and significantly associated with resilience, with the strongest association observed for use of emotion (*r* = 0.785) and the weakest for others’ emotion appraisal (*r* = 0.335). No significant associations were found between resilience and the sociodemographic and occupational variables examined.

**Discussion:**

The findings support an association between perceived emotional intelligence and resilience in the workplace and indicate differences in the magnitude of the associations across the dimensions of perceived emotional intelligence. However, given the cross-sectional design and the use of self-report measures, the results should be interpreted in associative rather than causal terms. Further research is needed to examine the relationship between the two constructs and their empirical distinctiveness.

## 1 Introduction

Emotional resilience has attracted growing interest in the field of occupational and organizational psychology in recent years (Hartmann et al., 2020). Understanding the factors that allow workers to adapt effectively to challenges in today’s workplace has become a priority of organizational research, especially in occupational environments defined by a high level of uncertainty, constant change, and intense emotional demands (Jiang et al., 2024).

Resilience has historically been associated with the ability to adapt positively to adverse situations. In this regard, Masten (2001) describes this adaptation as a set of adjustment processes that enable individuals to maintain and restore proper functioning despite exposure to threats that have a significant impact on their development and ability to adapt. Bonanno (2004) points out that resilience can also be seen as a relatively stable pattern of psychological functioning following exposure to events that might be considered traumatic. Furthermore, scientific literature in recent years has started to view resilience as a psychological resource that is important for managing daily life at work (Robertson et al., 2015). From this perspective, resilience can be considered the ability to maintain or restore adequate psychological functioning in the face of complex and stressful situations, enabling people to adapt to the changing circumstances of their current environment without compromising their well-being or performance (Luthar et al., 2000). In organizations, this ability is highly valued because professionals must simultaneously cope with intense work demands and interpersonal relationships that are often quite complex (Avey et al., 2009).

Parallel to the growing interest in resilience, emotional intelligence has gained significant prominence in organizational research as well. Based on the Ability Model developed by Mayer and Salovey (1997), this construct refers to people’s ability to perceive, understand, and regulate both their own emotions and those of others. These skills are especially well suited to work environments where social interaction and stress management are a regular part of the job. In fact, numerous studies have confirmed that people with higher emotional intelligence tend to cope more effectively with the emotional demands of work, and they display enhanced levels of psychological adjustment (Miao et al., 2017; Salovey et al., 1995). The present study assesses perceived emotional abilities using a self-report scale, a measurement approach that should be distinguished from performance-based ability tests (Joseph and Newman, 2010).

Drawing on these approaches, various studies have started to examine the relationship between emotional intelligence and resilience among the working population. In general, the results point to a positive association between the two concepts, suggesting that emotional skill can play a significant role in the ability to cope with struggles at work (Saklofske et al., 2012). According to Di Fabio and Saklofske (2018), emotional intelligence was significantly associated with resilience in a sample of workers, providing additional explanatory variance even when diverse personality models were considered. This finding is especially relevant, suggesting that emotional intelligence may provide key insights beyond personality traits, even though several authors have noted a certain conceptual overlap between the two domains (van der Linden et al., 2017). This similarity has led some scholars to assert that both of the constructs may share, at least partially, a common underlying mechanism related to broader processes of emotional adaptation, an issue that remains a subject of debate in the literature (Petrides et al., 2016; Petrides and Mavroveli, 2018).

Magnano et al. (2016) examined emotional intelligence, resilience, and achievement motivation among Italian workers. Their findings illustrate how these resources can be studied together, while the cross-sectional evidence does not establish temporal precedence. Theoretical work on emotion regulation and resilience offers possible accounts of their association (Tugade and Fredrickson, 2004), but the present study does not test a developmental mechanism.

The relationship between emotional intelligence and resilience has also been explored in theoretical frameworks that focus on psychological resources. One of the most influential constructs in organizational literature is the Job Demands-Resources model (Demerouti et al., 2001), which states that certain personal resources help employees cope with job demands and maintain a state of positive motivation (Bakker and Demerouti, 2014). From this perspective, both emotional intelligence and resilience can be considered personal resources that enable adaptation to complex work environments.

Chikobvu and Harunavamwe (2022) recently found an especially strong link between emotional intelligence and resilience among healthcare workers, while Mohseni Takalu et al. (2021) showed that emotional intelligence was not only positively associated with resilience among bank employees, but it also helped mitigate the negative impact of work-related stress.

Empirical literature has also observed this pattern in professional work settings defined by intense emotional demands. In the field of education, for example, significant associations have been reported between emotional intelligence and resilience in situations of prolonged stress (López-Angulo et al., 2022).

Another theoretical perspective considered relevant for interpreting this relationship is the Conservation of Resources Theory, or COR (Hobfoll, 1989). According to this approach, people strive not only to preserve, but to further develop the psychological resources they possess, in order to cope with the demands of their environment. In this conceptual framework, emotional intelligence is considered a personal resource that enables the development of other adaptive resources, including resilience. This idea is clearly illustrated in a study by Salas-Vallina and Elvira-Soria (2022), who found that emotional intelligence was indirectly associated with happiness at work through resilience. Similarly, Hayat et al. (2025) showed that both emotional intelligence and resilience mitigate the negative impact on the well-being of employees from certain workplace stressors, one of which is disrespect from co-workers.

In addition to its role as an adaptive resource, some studies consider resilience a possible mechanism linking emotional intelligence to various positive organizational outcomes. For example, Abualruz et al. (2024) found that resilience and psychological empowerment partially mediated the relationship between emotional intelligence and work engagement. Similarly, Castro Saucedo et al. (2018) revealed that emotional skill was one of the main predictors of resilience among professionals, who typically work under significant emotional pressure.

However, despite this body of converging evidence, it is worth noting that the relationship between emotional intelligence and resilience does not always follow the same pattern in all studies. In some cases, a direct association is revealed, while in others it becomes part of more complex models that include additional mediators or moderators. Jaggi and Gujral (2025) found that both emotional intelligence and resilience were positively correlated with mental health in the workplace, yet only emotional intelligence retained a significant predictive effect in the final model.

In summary, the preliminary literature review indicates certain limitations that justify further research in this area. For example, many studies use cross-sectional designs based on self-report measures, which makes it difficult to establish clear causal relationships (Creswell, 2014) and could even introduce potential bias associated with the measurement approach taken. Furthermore, there is considerable variation both in the instruments used to measure emotional intelligence and resilience, and in the occupational settings analyzed, which range from healthcare professionals to workers in the banking and educational sectors (Magnano et al., 2016). Although this diversity lends a degree of ecological validity to the results, it also highlights the need to continue examining these relationships in more heterogeneous workplace samples.

The primary aim was to examine the association between perceived emotional intelligence and individual resilience in a cross-sector sample of employees. A secondary, exploratory aim was to examine associations between resilience and sociodemographic and occupational characteristics. The study describes these associations without establishing causal direction or testing an intervention.

## 2 Materials and methods

### 2.1 Study design and participants

This study used a cross-sectional observational design (León and Montero, 2020).

The sample consisted of 103 employees working at the time of the study, aged between 19 and 64 years (*M* = 43.85, SD = 11.64). Participants were employed in 11 organizations based in Spain and belonging to different sectors of activity: renewable energy and electricity (11.7%), finance, investment and capital markets (10.7%), technology (ICT) (20.4%), agri-food industry (4.9%), education and training (20.4%), business and human resources services (7.8%) and tourism, health and wellness (24.3%). For reasons of confidentiality, no identifying information about the organizations is provided. The participants were selected using non-probabilistic convenience sampling, based on the available access to the organizations and the voluntary participation of their employees (Table 1).

**TABLE 1:** Characteristics of the sample.

Variable	Category	No.	%
Gender	Male	48	46.6
	Female	55	53.4
Age	19–64 (*M* = 43.85; SD = 11.64)		
Educational level	Primary education	4	3.9
	Secondary education, high school or vocational training	33	32.0
	Bachelor’s degree	34	33.0
	Postgraduate studies	32	31.1
Work experience	1–41 years (*M* = 20.23; SD = 10.83)		
Job level in the company	Operational or technical	52	50.5
	Middle management	34	33.0
	Senior management	17	16.5
Subordinate employees	None	57	55.3
	1–5	28	27.2
	6–10	8	7.8
	11–20	4	3.9
	21–50	5	4.9
	More than 50	1	1.0
Nationality	Spanish	99	96.1
	Other	4	3.9

Participants were eligible if they were employed, aged 18–65 years, and provided electronic informed consent. Responses were excluded if there were indications that difficulties understanding the questionnaire could compromise their validity.

Access to the survey was facilitated through collaboration with the human resources departments of the participating organizations, which helped distribute the online questionnaire to their employees.

Women accounted for 53.4% of the participants (*n* = 55), while men comprised 46.6% (*n* = 48).

As for educational level, 3.9% of participants had completed primary education, 32.0% had completed secondary education, high school or vocational training, 33.0% held a bachelor’s degree and 31.1% had completed postgraduate studies.

Regarding work experience, participants reported between 1 and 41 years of total experience (*M* = 20.23, SD = 10.83).

Operational or technical positions accounted for 50.5% of the sample, middle management for 33.0%, and senior management or executive positions for 16.5%.

Overall, 55.3% of participants had no subordinate staff; 27.2% supervised 1–5 employees, 7.8% supervised 6–10, 3.9% supervised 11–20, 4.9% supervised 21–50, and 1.0% supervised more than 50.

Regarding nationality, 96.1% of participants were Spanish, while 3.9% reported other nationalities.

### 2.2 Instruments

Data were collected through a Microsoft Forms questionnaire comprising sociodemographic and occupational questions and two standardized self-report instruments. Perceived emotional intelligence was assessed with the Spanish Wong and Law Emotional Intelligence Scale (WLEIS-S; Wong and Law, 2002; Extremera Pacheco et al., 2019), and individual resilience was assessed with the Spanish Resilience Scale-14 (RS-14; Wagnild, 2009; Sánchez-Teruel and Robles-Bello, 2015).

#### 2.2.1 Sociodemographic and socio-occupational questionnaire

The sociodemographic questions assessed gender, age, educational level, and nationality. Occupational questions assessed years of work experience, job level, number of subordinate staff, and company membership. Nationality was used for descriptive purposes. Associations with resilience were examined using ordinal representations of age, educational level, work experience, job level, and number of subordinate staff.

#### 2.2.2 Perceived emotional intelligence

Perceived emotional intelligence was assessed with the WLEIS-S (Extremera Pacheco et al., 2019), the Spanish adaptation of the WLEIS (Wong and Law, 2002). Its 16 items assess four dimensions: self-emotion appraisal (SEA), others’ emotion appraisal (OEA), use of emotion (UOE), and regulation of emotion (ROE). These scores represent participants’ perceptions of their emotional abilities rather than performance on an ability test.

Items were rated on a 7-point Likert scale from 1 (strongly disagree) to 7 (strongly agree). Item scores were summed to obtain a total score ranging from 16 to 112; higher scores indicate higher perceived emotional intelligence.

The total WLEIS-S score was used in the primary correlation analysis. Correlations between resilience and each of the four WLEIS-S dimensions were also examined to describe the pattern of associations at the dimensional level. Internal consistency of the total scale in this sample was high (Cronbach’s α = 0.888).

#### 2.2.3 Resilience

Resilience was assessed using the Resilience Scale-14 (RS-14; Wagnild, 2009) in its Spanish version adapted by Sánchez-Teruel and Robles-Bello (2015). The scale consisted of 14 items and assessed resilience as a psychological resource associated with people’s ability to adapt to and cope with adverse situations.

Items were rated on a 7-point Likert scale from 1 (strongly disagree) to 7 (strongly agree). Item scores were summed to obtain a total score ranging from 14 to 98; higher scores indicate higher resilience.

Table 2 summarizes the instruments. The Spanish RS-14 validation supported a unidimensional structure (Sánchez-Teruel and Robles-Bello, 2015), and the total score was therefore used in this study. Internal consistency was good (Cronbach’s α = 0.880).

**TABLE 2:** Instruments used.

Instrument	No. of items	Dimensions	Response range	α
WLEIS-S	16	SEA, OEA, UOE, ROE	1–7	0.888
RS-14	14	Unidimensional	1–7	0.880

### 2.3 Procedure

Data collection was conducted using an online questionnaire administered between June 2026 and September 2026. Access to the survey was facilitated through collaboration with the human resources departments of the participating organizations, which helped distribute the questionnaire link to the employees through their internal communication channels.

Participation was voluntary and confidential. Before completing the questionnaire, participants received information about the study objectives, voluntary participation, and data processing, and provided electronic informed consent.

The questionnaire was self-administered online. It first assessed sociodemographic and occupational characteristics and then perceived emotional intelligence and resilience. All questionnaire items required a response, so submitted questionnaires contained no missing item responses.

Submitted responses were checked for errors and inconsistencies. No invalid or out-of-range values were identified.

The study was conducted in accordance with the Declaration of Helsinki (World Medical Association, 2013) and received ethics approval (Code 854/24/107). Participants provided electronic informed consent before completing the questionnaire.

### 2.4 Data analysis

Analyses were performed using IBM SPSS Statistics, version 29. Composite scores were checked for scoring errors and out-of-range values before analysis.

Descriptive statistics included means, standard deviations, observed ranges, skewness, and kurtosis. Distributional assumptions were assessed using Kolmogorov–Smirnov tests and visual inspection of histograms and Q–Q plots. The tests were not statistically significant for either total score (*p* > 0.05), and graphical inspection supported the use of parametric analyses.

Pearson correlations were used to examine associations between total perceived emotional intelligence and resilience and between each of the four WLEIS-S dimensions and total resilience. These analyses describe differences in observed correlation magnitudes; they do not constitute formal tests of differences between dependent correlations.

A one-way ANOVA assessed differences in resilience between the 11 companies, with company membership as the grouping variable. Levene’s test assessed homogeneity of variances. A random-intercept model was also used to estimate ICC(1), the proportion of variance in resilience attributable to differences between companies. These analyses examined clustering in resilience; they did not directly assess clustering in emotional intelligence or the within-company association between the two constructs.

Spearman correlations were used to examine associations between resilience and ordinal sociodemographic and occupational variables: age, educational level, work experience, job level, and number of subordinate staff.

An independent-samples *t*-test examined differences in resilience by gender, with Welch’s test used if the equal-variance assumption was not met. Cohen’s d was used to describe the standardized mean difference. Tests were two-sided, with a significance threshold of *p* < 0.05. Sociodemographic, occupational, and subscale analyses were treated as exploratory.

## 3 Results

### 3.1 Descriptive statistics and normality

Perceived emotional intelligence had a mean score of 89.59 (SD = 10.15; range = 62–112), while resilience had a mean score of 82.48 (SD = 8.68; range = 59–98). The values for skewness (EI = −0.36; resilience = −0.43) and kurtosis (EI = −0.07; resilience = −0.22) fell within acceptable ranges, indicating approximately symmetrical distributions.

Kolmogorov–Smirnov tests were not statistically significant for either total score (*p* > 0.05). Histograms and Q–Q plots showed approximately normal distributions without conspicuous extreme values.

### 3.2 Common method bias assessment

An unrotated principal components analysis of all 30 WLEIS-S and RS-14 items was used for Harman’s single-factor assessment. Sampling adequacy was acceptable (KMO = 0.845), and Bartlett’s test was significant, χ^2^(435) = 1967.450, *p* < 0.001. Five components had eigenvalues greater than 1. The first component accounted for 35% of total variance. Although this was below the frequently cited 50% threshold, the test cannot establish the absence of common method bias or demonstrate discriminant validity (Podsakoff et al., 2003).

### 3.3 The relationship between emotional intelligence and resilience

Total perceived emotional intelligence and resilience were strongly and positively correlated, *r*(101) = 0.762, *p* < 0.001, 95% CI [0.67, 0.83] (Table 3). The squared correlation was 0.581, corresponding to approximately 58.1% shared variance between the observed scores.

**TABLE 3:** Descriptive statistics and the correlation between emotional intelligence and resilience.

Variable	M	SD	1	2
Perceived emotional intelligence	89.59	10.15	–	
Resilience	82.48	8.68	0.762***	–

***p < 0.001.

As a complement to this finding, associations between the total resilience score and the four dimensions of perceived emotional intelligence were also analyzed (Table 4). All of these dimensions showed positive and statistically significant correlations with total resilience (*p* < 0.001).

**TABLE 4:** Correlations between resilience and the dimensions of perceived emotional intelligence.

WLEIS dimension	r with resilience	P
Self-emotion appraisal (SEA)	0.661	<0.001
Others’ emotion appraisal (OEA)	0.335	<0.001
Use of emotion (UOE)	0.785	<0.001
Regulation of emotion (ROE)	0.567	<0.001

The correlation with resilience was numerically largest for use of emotion (*r* = 0.785), followed by self-emotion appraisal (*r* = 0.661), regulation of emotion (*r* = 0.567), and others’ emotion appraisal (*r* = 0.335). These are descriptive comparisons of the observed correlations; differences between the correlations were not formally tested.

### 3.4 Differences in resilience by company

Resilience did not differ significantly between companies, *F*(10, 92) = 0.941, *p* = 0.500 (Table 5). Levene’s test was not statistically significant (*p* = 0.153). The sample effect size was *η*^2^ = 0.093, corresponding to approximately 9.3% of the total sum of squares in the ANOVA. This sample effect size is distinct from the random-intercept ICC, which was estimated to be approximately zero. The latter suggests little estimated between-company variance in resilience, but an estimate near zero does not establish that observations are independent, particularly with only 11 companies.

**TABLE 5:** Analysis of variance (ANOVA) of resilience by company.

Source	F	df	P	*η* ^2^
Company	0.941	(10, 92)	0.500	0.093

### 3.5 Sociodemographic variables and resilience

No significant associations were found between resilience and work experience, age, educational level, job level, or the number of subordinate staff (Table 6).

**TABLE 6:** Spearman’s correlation between sociodemographic variables and resilience.

Variable	r_*s*_	P
Age	0.123	0.218
Educational level	0.112	0.260
Years of work experience	0.119	0.232
Job level in the company	0.159	0.110
Number of subordinate staff	0.124	0.212

### 3.6 Differences in resilience by gender

Resilience did not differ significantly between men and women, *t*(101) = −1.437, *p* = 0.154 (Table 7). The reported absolute standardized mean difference was —d| = 0.28. A nonsignificant result does not establish equivalence between the groups.

**TABLE 7:** Results of *t*-tests for independent samples: differences in resilience by gender.

Variable	t	df	P	—d—
Resilience	−1.437	101	0.154	0.28

## 4 Discussion

This study identified a strong positive association between perceived emotional intelligence and self-reported resilience in a cross-sector sample of employees in Spain. The findings are consistent with research treating these constructs as related personal resources in occupational settings. However, the measures assessed general individual characteristics rather than adaptation to a specific workplace adversity, and workplace well-being and performance were not measured.

The magnitude of the correlation warrants caution. Previous studies have reported positive associations between emotional intelligence and resilience (Di Fabio and Saklofske, 2018; Magnano et al., 2016), but shared item content and self-report response tendencies may contribute to the association in this study. Harman’s assessment indicated that the first component accounted for 35% of item variance. This finding neither rules out common method bias nor determines how much of the correlation reflects that bias. Social desirability, general positive self-evaluation, and conceptual overlap remain plausible explanations for part of the shared variance (Podsakoff et al., 2003). The present analyses do not establish the empirical distinctiveness of the constructs.

One possible interpretation is that perceived emotional abilities and resilience reflect related aspects of adaptation. Emotion appraisal and regulation may be relevant to coping with work demands, as suggested by theoretical accounts of emotional intelligence and emotion regulation (Mayer et al., 2016; Gross, 2015). These mechanisms were not measured directly and therefore remain interpretations rather than demonstrated pathways.

In education, positive associations between emotional intelligence and resilience have been reported (López-Angulo et al., 2022). Related literature also examines emotional intelligence in relation to teacher burnout (Mérida-López and Extremera, 2017). These outcomes should be distinguished: evidence concerning burnout does not itself demonstrate a relationship with resilience.

In healthcare, emotional intelligence and resilience have been examined together in relation to work engagement (Chikobvu and Harunavamwe, 2022). Such research provides occupational context for the present association, while differences in samples, measures, and outcomes limit direct comparisons.

The findings also fit within positive organizational psychology, which examines personal resources in relation to well-being and adjustment at work (Luthans et al., 2007). Salas-Vallina and Elvira-Soria (2022) investigated emotional intelligence and resilience in relation to happiness at work. The present study examined the association between the two resources without measuring happiness, adjustment, or performance outcomes.

The broaden-and-build theory proposes that positive emotions contribute to the development of psychological resources (Fredrickson, 2001; Tugade and Fredrickson, 2004). Conservation of Resources theory similarly emphasizes the preservation and accumulation of resources under demanding conditions (Hobfoll, 1989). These frameworks offer possible interpretations of the association, but their proposed processes were not tested here.

The high correlation also raises questions about construct boundaries. Sarrionandia et al. (2018) examined resilience as a statistical mediator of the relationship between emotional intelligence and perceived stress. Such models can help formulate hypotheses about related resources, but do not by themselves establish that the constructs are psychometrically distinct.

Conceptual distinctions between performance-based emotional intelligence and self-reported emotional abilities are particularly relevant. Trait emotional intelligence concerns emotional self-perceptions and overlaps with aspects of personality (Petrides et al., 2016; Petrides and Mavroveli, 2018). The WLEIS-S used here assesses perceived emotional abilities and should not be interpreted as an objective measure of emotional performance.

Research has documented overlap between emotional intelligence and broader personality factors (van der Linden et al., 2017), while Di Fabio and Saklofske (2018) reported an incremental contribution of emotional intelligence to resilience beyond personality measures. Our bivariate analyses did not adjust for personality and cannot establish incremental validity. More broadly, work on resilience assessment underscores the need for careful construct definition and measurement (Prince-Embury et al., 2017).

The dimensional results provide additional descriptive information: the observed association with resilience was largest for use of emotion and smallest for others’ emotion appraisal. The total score summarizes the overall association, whereas subscale scores reveal variation within perceived emotional intelligence. Neither these correlations nor the use of a total score establish that the dimensions have interchangeable associations with resilience.

Within the Job Demands–Resources framework, emotional intelligence and resilience may be conceptualized as personal resources (Demerouti et al., 2001; Bakker and Demerouti, 2014; Bakker et al., 2023). Because job demands and organizational resources were not measured, the present findings are compatible with this framework but do not test it.

Associations with an outcome may also differ when emotional intelligence and resilience are modeled together. Jaggi and Gujral (2025), for example, found that both were associated with mental health, but only emotional intelligence retained a statistically significant association in their joint regression model. This result concerns their associations with mental health; it does not demonstrate a nonlinear or absent relationship between emotional intelligence and resilience. Related occupational research also examines resilience in relation to stress and business outcomes (Shatté et al., 2017).

The random-intercept model yielded an ICC close to zero for resilience. This estimate suggests little between-company variation in that outcome in this sample, but it does not confirm independence for all variables or validate the pooled correlation as a within-company association. With 11 companies and potentially uneven cluster sizes, uncertainty in the variance-component estimate remains relevant (Bliese, 2000).

No statistically significant associations were detected between resilience and the sociodemographic or occupational variables examined. These findings describe the available sample and should not be interpreted as evidence that such characteristics are unrelated to resilience in the wider working population.

The cross-sector sample adds evidence on the association between perceived emotional intelligence and resilience among employees. However, convenience recruitment and the small sample limit generalizability. The study also does not establish that emotional intelligence training would increase resilience; intervention studies would be needed to evaluate that possibility.

### 4.1 Limitations

Several limitations affect the interpretation and generalizability of the findings.

First, the cross-sectional design precludes causal and temporal inferences. Neither the direction of the association nor a mediating process can be established from these data. Longitudinal studies are needed to evaluate the temporal hypotheses proposed in earlier research (Wang et al., 2023).

Second, all psychological measures were self-reports collected at one time point. Common method variance, social desirability, and overlapping content may inflate observed associations (Podsakoff et al., 2003; Petrides et al., 2016). Harman’s assessment is not a sufficient test of these explanations, and no direct assessment of discriminant validity was reported. High reliability coefficients do not resolve this limitation.

Third, the study did not control for personality, perceived stress, or other potentially related characteristics. Prior evidence on the joint roles of emotional intelligence and resilience (Di Fabio and Saklofske, 2018; Sarrionandia et al., 2018; Jaggi and Gujral, 2025) cannot substitute for adjustment or construct-validity analyses in this sample.

Fourth, convenience sampling, voluntary participation, and recruitment through human resources departments may introduce selection bias. The sample was small and spanned multiple sectors; representation of sectors does not imply representativeness of the working population. Nonsignificant exploratory findings may reflect limited precision, especially in small subgroups.

Fifth, only 11 organizations were represented. The ICC for resilience does not exclude clustering in emotional intelligence or uncertainty in the pooled association. Cluster sizes, variance components, and uncertainty estimates would help assess this limitation (Bliese, 2000).

Finally, subscale correlations were compared descriptively, without formal tests of their differences. Several exploratory comparisons were performed, so these findings should be interpreted cautiously. The RS-14 assessed general individual resilience; exposure to workplace adversity, workplace-specific adaptation, well-being, and performance were not assessed.

### 4.2 Future research directions

Future research should clarify the temporal relationship, measurement boundaries, and occupational relevance of these constructs.

Longitudinal studies could examine whether earlier perceived emotional intelligence is associated with later resilience, or vice versa, while accounting for baseline levels and relevant covariates. Repeated measurement would also help examine changes in these resources across working conditions (Masten and Motti-Stefanidi, 2020; Wang et al., 2023).

Measurement studies should combine self-reports with performance-based emotional intelligence measures, independent ratings, or behavioral outcomes. Appropriately powered latent-variable analyses could evaluate whether distinct constructs account for the data better than a shared factor. Such approaches would address construct overlap more directly than Harman’s single-factor assessment.

Larger samples from more organizations could support multilevel analyses of personal resources alongside job demands, leadership, organizational climate, and perceived support. This would permit direct tests of contextual hypotheses drawn from the Job Demands–Resources framework (Bakker and Demerouti, 2017).

Intervention research could evaluate whether emotional intelligence training is followed by improvements in resilience and occupational outcomes. Evidence that emotional intelligence can be trained (Mattingly and Kraiger, 2019) does not establish that such training increases resilience. Controlled studies should measure both constructs and assess whether any changes are distinct from shared response tendencies.

Together, these approaches would provide a more precise account of the association between perceived emotional intelligence and resilience across occupational settings.

Recent Frontiers editorials emphasize learning, training, and supportive environments in discussions of resilience and employability (Smaliukienè et al., 2024, 2025). These perspectives motivate future intervention research; they do not establish intervention effects in the present sample.

## Data Availability

The data supporting the findings of this study are not publicly available because they contain sensitive participant information, and participants did not consent to public sharing of their data. A de-identified minimum dataset may be made available by the corresponding author upon reasonable request, subject to applicable ethical and data protection requirements.
